# Safe and usable ensemble formation for networked medical devices: a comparative study of 5G, NFC and pop-up pairing

**DOI:** 10.1007/s11548-026-03618-y

**Published:** 2026-04-13

**Authors:** Okan Yilmaz, Simon Kißmann, Marlene Goschkowski, Philipp Schollmaier, Frank Beger, Dario Wieschebrock, Philipp Feodorovici, Jan C. Arensmeyer, Detlef Fleer, Dubravka Maljevic, Klaus Radermacher, Armin Janß

**Affiliations:** 1 Chair of Medical Engineering, Helmholtz-Institute for Biomedical Engineering of the RWTH Aachen University, Pauwelsstraße 20, 52074 Aachen, Germany; 2 Beger Design, Im Zollhafen 12, 50678 Cologne, Germany; 3 SurgiTAIX AG, Kaiserstraße 100, 52134 Herzogenrath, Germany; 4 Department of Thoracic Surgery, University Hospital Bonn, 53127 Bonn, Germany; 5 Bonn Surgical Technology Center (BOSTER), University Hospital Bonn, 53227 Bonn, Germany; 6 OR Management, University Medicine Essen, 45147 Essen, Germany; 7 Medical Engineering, BG Kliniken IT-Services gGmbH, 10178 Berlin, Germany

**Keywords:** Digital operating room, Human-machine interaction, Ensemble formation, Interoperability, Service oriented device connectivity, SDC

## Abstract

**Purpose:**

Incorrectly formed medical device ensembles can cause therapy to be performed at the wrong site or display safety-critical data from the wrong patient. This early, exploratory study investigates which wireless ensemble-creation techniques minimize risk while remaining safe and usable in intraoperative workflows. The goal is to identify the most promising methods among 5G, NFC, and pop-up pairing.

**Methods:**

Eight techniques (5G proximity/location, pop-up pairing, QR Code, Near-Field Communication (NFC) Tag and Reader, device-ID copying, manual selection, device interaction, and SDC context lookup) and five clinical situations (patient bedside, transport, prep room, operating room (OR), post OR) were examined. Five interoperability and clinical experts rated the importance of risk- and usability-related criteria for each situation, as well as how well the criteria are being fulfilled by the different methods. The criteria weights and the method-specific ratings were combined in a quantitative decision matrix to identify the three most promising approaches, which were implemented in a click-dummy prototype and evaluated through formative testing with two clinicians.

**Results:**

The analysis rated 5G, NFC, and pop-up pairing the highest when combining all categories. Subsequent formative tests with clinicians from the University Hospital Bonn support technical feasibility and highlight clear trade-offs. In this small sample (*n* = 2), NFC was perceived as the safest and most preferred technique. 5G offered speed and automation potential, but raised critical concerns regarding accuracy. Pop-up pairing was considered flexible yet error-prone when multiple devices entered pairing mode simultaneously.

**Conclusion:**

This is a systematic comparison of wireless SDC-ensemble techniques. Early clinical feedback favors NFC for its perceived safety, while 5G and pop-up pairing approaches require additional refinements to mitigate residual risks. As one participant pointed out: “I prefer a reliable and slower over an automated but error-prone method”.

## Introduction

Networked medical devices and the number of complex technical systems are increasing in modern ORs (operating rooms) [1]. Intelligent data exchange, remote control, and closed-loop assistance systems promise increased safety and workflow efficiency [2].

The ISO/IEEE 11073 Service-Oriented Device Connectivity (SDC) standard family formalizes this interoperability by providing a standardized method for medical devices to communicate with each other [3]. Two devices, which intend to collaborate and perform a distributed system function, must first establish an “SDC Participant Ensemble” based on available context information, such as patient identity and physical location [4]. This is a crucial requirement for developing and using central SDC Workstations within an interconnected OR.

While hard-wiring solutions mitigate cross-room interference, the growing adoption of wireless solutions potentially extends connectivity beyond a single operating theatre, thereby increasing the risk of erroneous ensemble formation. Less wiring has the advantage of removing tripping hazards, reducing risks induced by accidental disconnections, and supporting flexibility of more mobile solutions [5].

In high-workload and time-pressured scenarios, the ensemble pairing process must be safe, usable, and intuitive. If an operating room table or infusion pump were inadvertently associated with the wrong control unit, the table movement or the wrong delivery of medication could lead to catastrophic harm. Existing literature and standards require that such medical device ensembles are flexible, yet no published study has proposed validated methods for (wireless) SDC ensemble creation [4, 6].

Martínez and González demonstrated a wireless ISO/IEEE 11073‑compliant solution using patient identification via barcodes or RFID, enabling secure association between Device IDs and Patient IDs and reducing identification errors [7].

Eight pairing methods were screened in this study:

*5G proximity/location:* Users can see in a Workstation the absolute position (~ 1 m accuracy) and the proximity (close, moderate, far) to the closest antenna of a medical device. The user must manually choose the correct device based on distance and device type/name. [8, 9]

*Pop-up pairing*: Users can start a matching action on a Workstation and must confirm it on the medical device. Both show unique, identifiable information.

*NFC Tag and Reader*: Users use an NFC Scanner, and devices and workstations have NFC Tags. They tap the Workstation and the medical device once.

*SDC context lookup*: Medical devices may have additional context properties, such as the patient to which they are connected. Devices that are moved together can assume their new location by copying it from other devices with the same patient context in that new location. This technique is called “automatically inferred location ensemble,” as described in [4].

*QR Code and Reader*: Users use a QR Code Scanner to scan printed and glued QR Codes from medical devices.

*Device-ID copying*: Users read a device’s unique ID from its screen and enter it into a Workstation.

*Device interaction*: User goes to a Workstation and connects to all medical devices of a certain type. By interacting with the medical device and changing its property (e.g., moving the OR table), one can identify the correct device.

*Manual selection*: A user can see all medical devices on the network and choose freely which device to connect to.

Given the lack of prior work on wireless SDC ensemble formation, we chose an early-stage, exploratory design. The goal was not to provide statistically generalizable evidence, but to screen promising techniques and gain initial qualitative feedback.

More specifically, this exploratory study addressed the following research questions: Among eight candidate methods, which wireless ensemble-formation techniques achieve the most favorable balance between safety‑ and usability‑related criteria across five clinical scenarios?

## Materials and methods

### Study design

We conducted a multi-criteria decision analysis with an expert panel to rank eight ensemble-formation techniques, involving five experts. This was followed by a formative think-aloud usability test of the three top-ranked methods with two clinicians.

### Participants

We established an expert panel comprising two clinicians and three interoperability experts (n = 5). The experts were members of the working group “ensemble formation” of the German research project CLOUD56. The eligibility criteria for experts were familiarity with medical device workflows and interoperability standards. The end users for the formative usability testing were two clinicians from University Hospital Bonn, Bonn, Germany. The panel was intentionally composed to cover both clinical and technical perspectives.

### Peri-operative scenarios

Workshops at the University Hospital Bonn identified five scenarios in which ensembles are typically created or updated. Those scenarios are patient bedside, transport, preparation room, operating room (OR), and post-OR.

### Criteria catalogue and weighting

With in-house workshops and discussions, nine criteria were extracted from ISO 14971, ISO 9241-110, IEC 62366-1, as well as categories that are essential for commissioning, such as automation potential `10–12]. Each panelist assigned an importance/weight-score of 1–5 (5 = most important) to each scenario.

### Technique screening and rating

The same panel of experts conducted multiple workshops, discussing each technique against the nine criteria. When a consensus was reached (by majority vote), a rating was assigned on a 1–5 scale (5 = best).

### Multi-criteria decision analysis

To determine the importance of each category, ratings were collected on 5‑point Likert‑type scales (1 = not important, 5 = extremely important). The ratings were averaged. For each technique t and scenario s, we calculated a weighted score for each (technique, scenario) ${S}_{t,s}$, where ${w}_{s,c}$ is the scenario specific weight and ${{\boldsymbol{r}}}_{{\boldsymbol{t}},{\boldsymbol{c}}}$ the technique specific rating.

$$ S_{t,s} = \sum\limits_{c = 1}^{9} {W_{S,C} r_{t,c} } $$

### Formative usability test

**Fig. 1:**
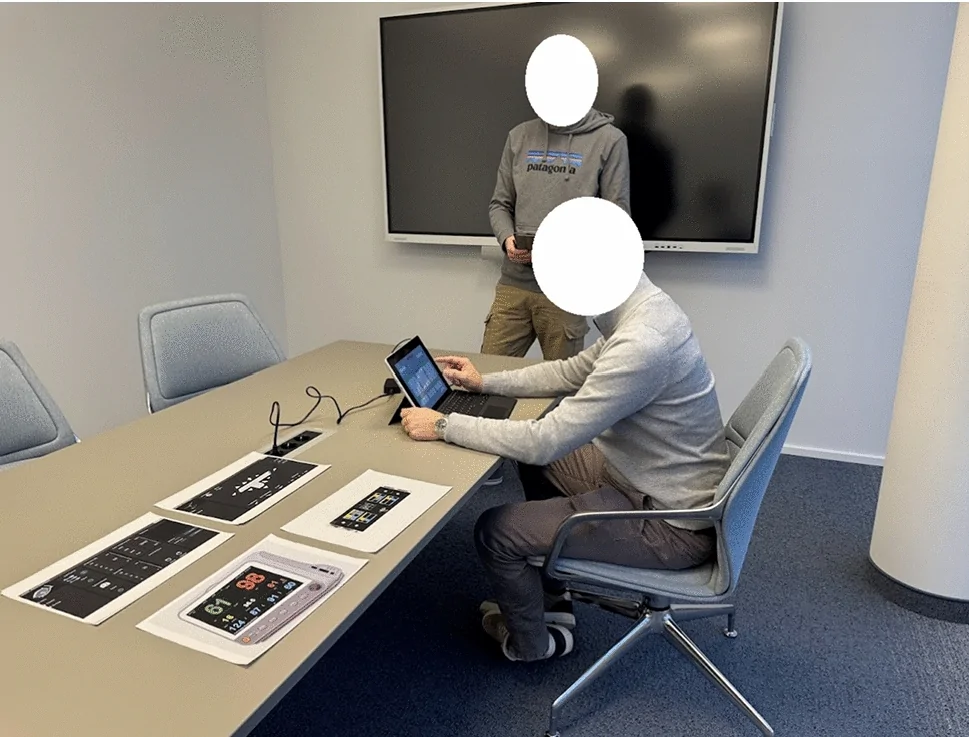
Setup of the usability study: the participant is sitting while the moderator reads the task. Medical devices are shown as printed papers on the table

Two clinicians from the University Hospital Bonn conducted a user-centered study by testing the click-dummy and creating various SDC device ensembles using the top three-rated techniques. We employed the think-aloud method and observed participants as they performed tasks, including selecting the correct device and pairing using different techniques (Fig. 1**)**. [10]

## Results

### Clinical scenarios

**Fig. 2:**
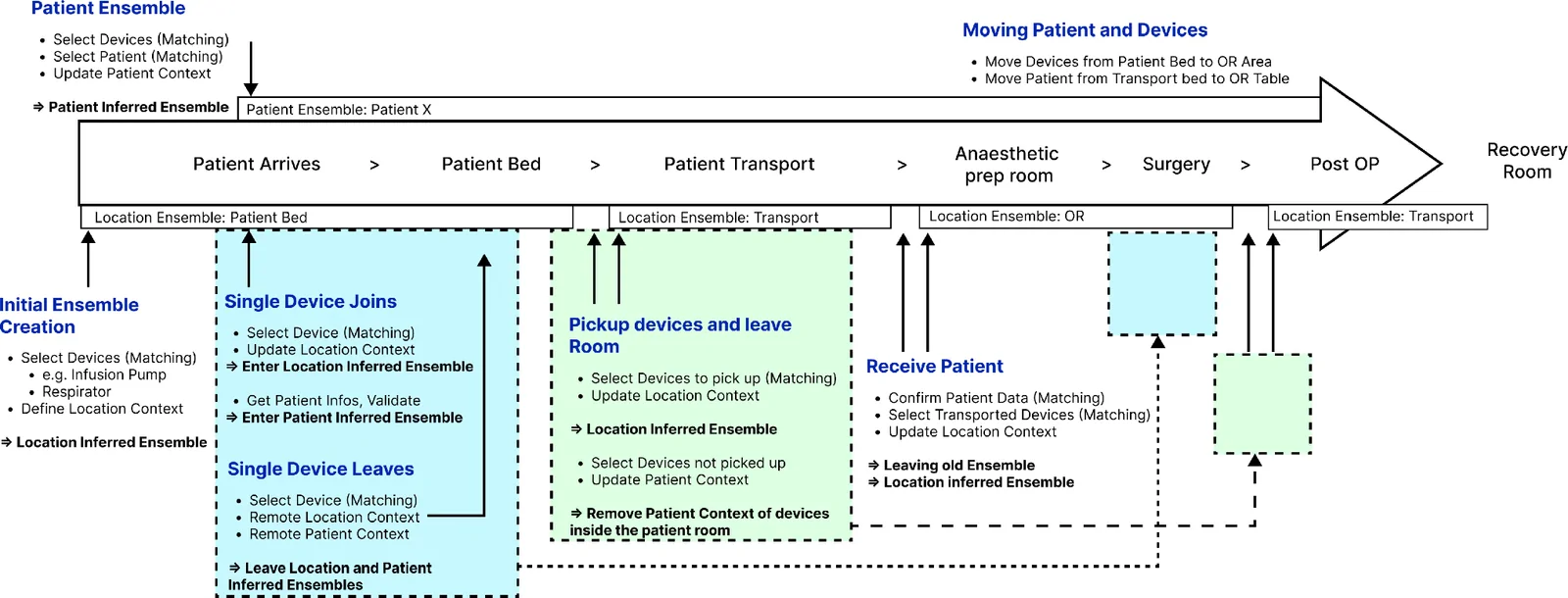
Flowchart showing the different times at which SDC ensembles change

Workflow analyses at the University Hospitals Aachen and Bonn confirmed five key scenarios in which an SDC ensemble must be created or updated (see Fig. 2**)**. Ideally, a patient ensemble is created on admission and remains valid throughout the hospital stay, whereas location context changes whenever the patient moves.

### Multi-criteria decision analysis

The highest importance was allocated to probability of errors (22.7), time effort (21.5), and ease of use (20.7), while the lowest was additional equipment (12.8) and additional cleaning effort (12.8). The top 3 categories from Table 1 align with the attributes (efficiency, effectiveness, and user satisfaction) [11].

**Table 1:**
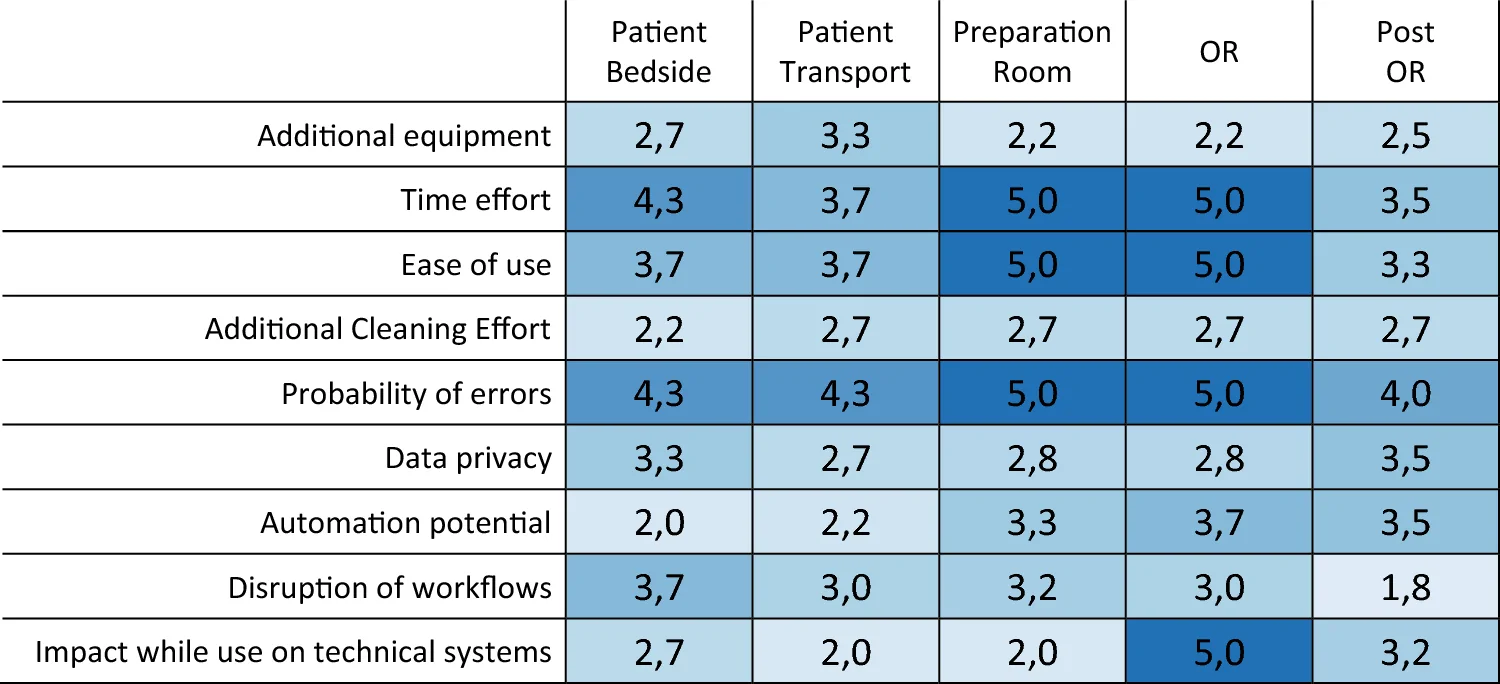
Importance ratings of nine usability and risk criteria for five clinical situations (5: extremely important, 3: moderately important, 1: not important)

Table 2 shows eight wireless ensemble formation techniques on the X-axis and nine usability and risk criteria on the Y-axis, with each cell containing a five-point rating. Some methods have residual risks that could be mitigated through additional refinements or by combining methods. These were not included.

**Table 2:**
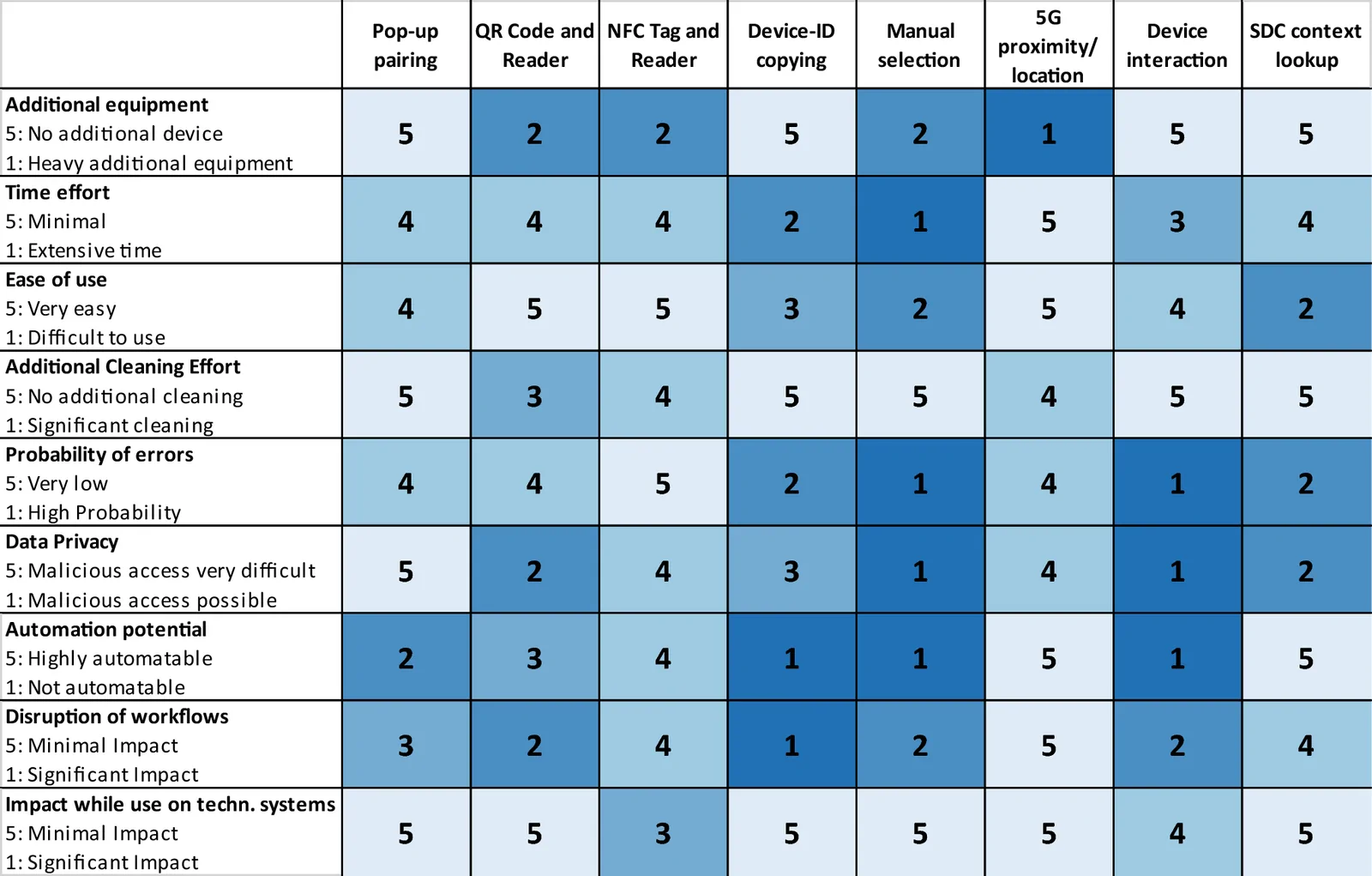
Ratings of eight ensemble formation techniques according to nine usability and risk criteria

**Fig. 3:**
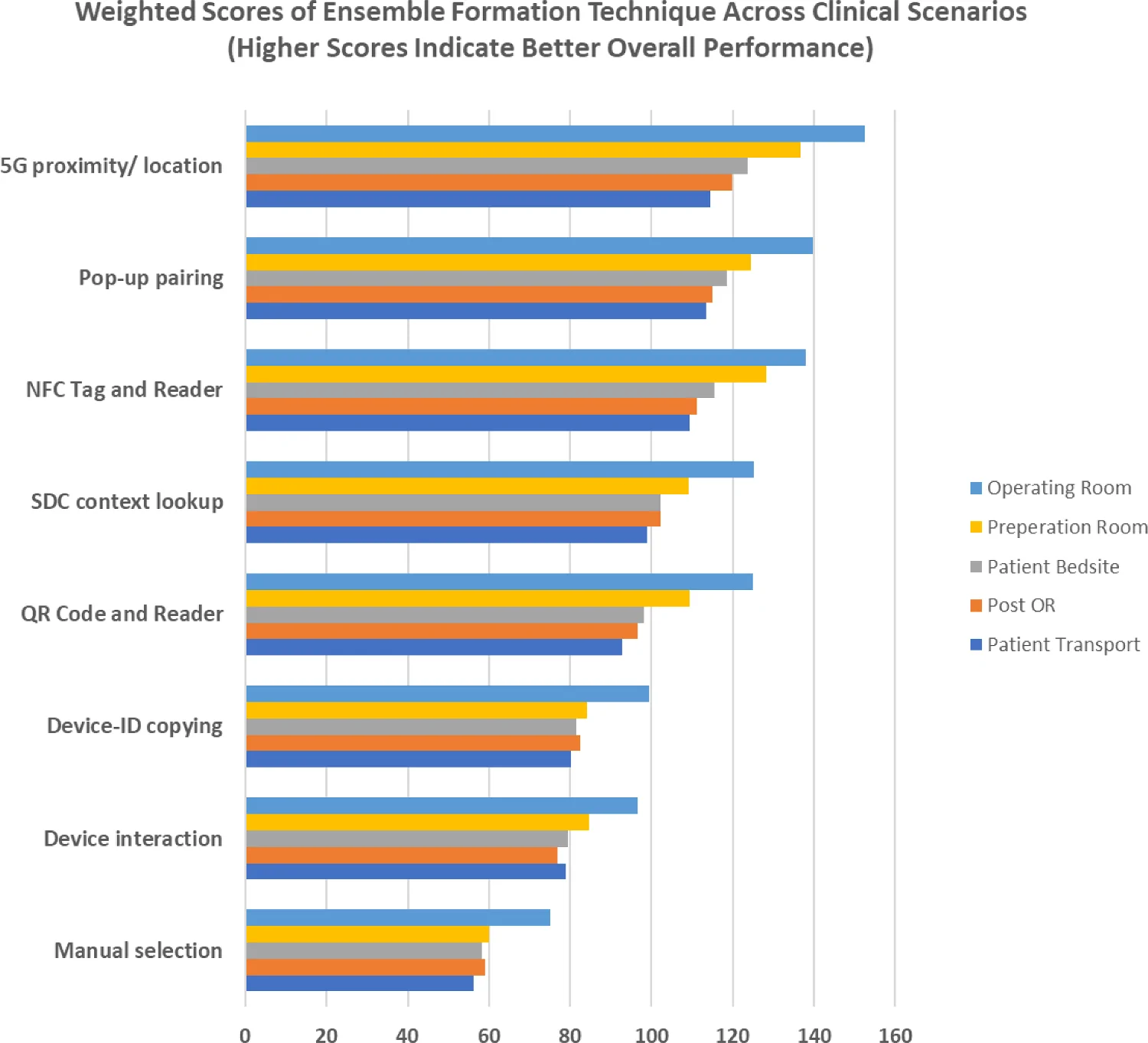
Weighted Scores of ensemble formation techniques across clinical situations

Figure 3 visualizes the results, sorted by the sum of ratings across all clinical scenarios. A higher score indicates a better overall performance. The absolute magnitudes of these scores are not meaningful on their own and should only be interpreted in relation to one another. The methods are sorted in descending order in Fig. 3. In a sensitivity analysis, doubling the weights of the safety‑ and security‑related criteria (Probability of errors, Data privacy, Impact while use on technical systems) or any other single criterion did not change the top‑3 ranking.

### Prototype implementation

We implemented and evaluated the three top-ranked techniques in a Figma (v125.5.6) click-dummy. Since we are in an early prototype stage, we focused on the methods that already look best on paper. Implementing click-dummies for all scenarios would be very time-consuming.

The participants used a touchscreen device (Microsoft Surface Pro 9 5G, 13") to interact with the ensemble manager. On the table, printed paper prototypes represented an OR-Light, a patient monitor, an OR-Table, and a High-Frequency electrosurgical device.

**Fig. 4:**
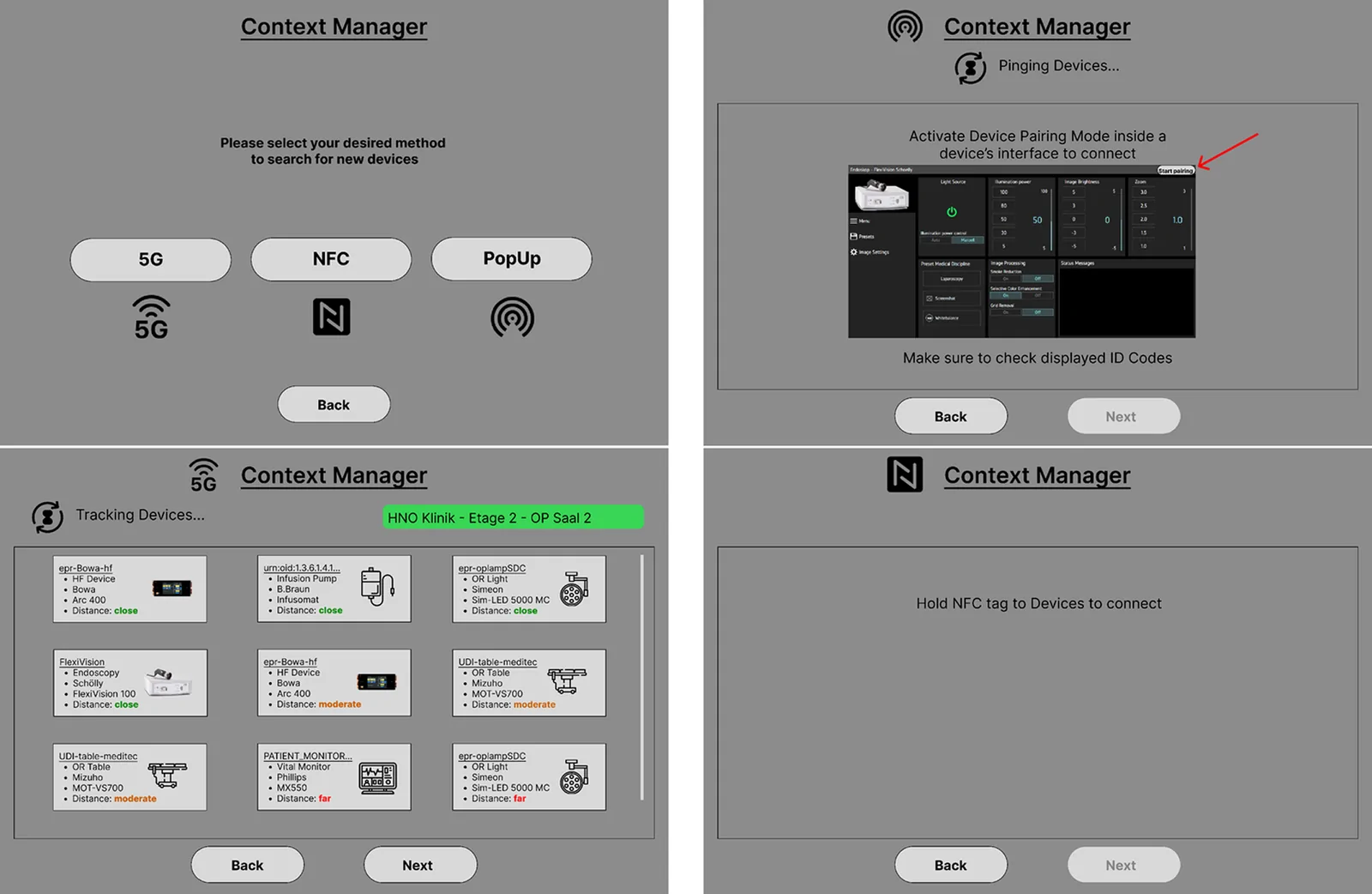
Screenshots of the developed context manager for the ensemble creation process. Top Left: window, which allows the participant to choose the method to be used. Top Right: pop-up pairing describing how to start the device pairing process. Bottom Left: 5G Method, showing a list of tracked devices and detailed information. Bottom right: NFC method, prompting the user to hold the NFC tag to a device

The prototype shown in Fig. 4 emulates the graphical user interface of an SDC context manager tablet and implements the complete workflow for creating an ensemble, including device discovery, device selection, assignment of patient & location contexts, and the final confirmation step. The prototype implemented three alternative interaction flows. For the *5G proximity/location* technique, the “discover” screen remained blank for two seconds before populating with devices sorted by distance (close/moderate/far) to mimic real-time positioning latency. In the *NFC Tag and Reader* technique, no devices were shown until the participant touched the device using an NFC tag. For the *Pop-up pairing* technique, participants first had to press the pairing button on the printed target device. The tablet then displayed two candidate devices, each accompanied by a four-digit code, and the user confirmed the ensemble by matching the codes.

### Formative evaluation results

The feedback given during the formative study is listed below and can be divided into four categories, three of which relate to specific techniques and the fourth to general aspects:

- **5G proximity/location**
  o “Is the distance reliable?”
  o “If there is a chance for an error, it will happen.”
  o “Using 5G everywhere with private networks seems expensive.”
  o “Sorting by distance is a great feature”
  o “Everything far should be excluded from the view”
- **Pop-up pairing**
  o “There is a high risk due to blindly accepting the pop-up.”
  o “I have to run back and forth all the time.”
  o “Not 100% safe.”, “Comparing code shown on both screens provides additional safety.”
- **NFC Tag and Reader**
  o “I prefer a reliable and slower over an automated but error-prone method.”
  o “Touching 5 devices with an NFC tag is much better than clicking around in lists on a touchscreen.”
  o “A mobile reader can/will be lost.”
  o “We have company-issued phones. Can those be used instead of RFID chips?”
  o “NFC seems to be a safe, quick, intuitive, and cost-effective solution.”
- **General comments**
  o “For every profession (anesthetists, surgeons, surgical assistants), a different set of devices is relevant. Filtering according to profession would be useful.”
  o “Non-Moveable devices such as room equipment should be shown as such”
  o “An additional confirmation of the ensemble is unnecessary.”
  o “The used colors are irritating”, “I like the pictograms of the devices.”

## Discussion

This work represents a systematic comparison of different wireless ensemble-formation techniques for ISO/IEEE 11073 SDC-enabled medical devices across the perioperative pathway. Using a two-stage method (multi-criteria decision analysis followed by formative usability testing), we screened different techniques and chose three approaches: 5G proximity/location, NFC tag & reader, and pop-up pairing. Those were selected out of an initial set of eight candidates and balanced safety-related and usability-related requirements.

5G positioning achieved the best weighted risk/usability score in the decision, owing to its strong automation potential and minimal workflow disruption, but raised clinician’s concerns regarding positioning accuracy and infrastructure costs. 5G location services indoors (such as mobile objects on a factory floor) can reach sub-meter accuracy [12]. However, reflections from metallic OR surfaces and the regulatory overhead of private 5G deployments introduce uncertainty and additional risks. Without robust mitigation, such as multi-modal confirmation, the risk of cross-room or cross-patient pairing remains.

NFC was rated as the safest by experts and end-users alike, but it requires a reader/tag and introduces manual effort. The limited range and mandatory physical action prevent man-in-the-middle attacks [13].

Pop-up pairing was perceived as moderately efficient and flexible, yet its reliance on visual code comparison and the possibility of “blind acceptance” make it more error-prone in busy OR environments.

Patient-safety considerations are essential when creating SDC ensembles and must therefore be carefully integrated into existing risk‑management processes for networked medical devices and IT security regulations. The safety and unambiguity should not be traded in for speed or a better user experience. Explicit confirmation techniques (NFC, QR) slow the workflow, but give staff clear control, which participants in our formative study valued and ​summarized “I prefer a reliable and slower over an automated but error-prone method”.

With this analysis, we were able to provide an early, exploratory answer to our central research question, which wireless ensemble-formation techniques achieve the most favorable balance between safety‑ and usability‑related criteria across five clinical situations.

### Limitations and future work

The study is exploratory and based on a very small sample (five experts, two of whom were clinicians). The resulting rankings and qualitative impressions should therefore be interpreted as indications rather than generalizable evidence about safety or usability. Future work will be required to validate the present findings in larger formative usability studies that involve actual medical devices and realistic OR conditions.

It should also investigate hybrid workflows that combine different ensemble formation techniques (for example, 5G positioning with NFC), creating a balance between automation and safety. Further refinement of the context-manager software should enable the automatic display of profession-specific devices, thereby reducing cognitive load. Future prototypes should explore embedding NFC hardware directly into devices and use clinicians’ smartphones as readers, accompanied by cost–benefit analyses that consider existing equipment and cleaning demands. Finally, the multi-criteria decision matrix should be extended with additional and increased weights for security.

## Conclusion

Among eight candidate techniques for forming SDC ensembles, our two-stage evaluation singled out three viable options. Clinicians favored NFC for its clear, deliberate confirmation step, while 5G proximity/location excelled in speed but still requires accuracy safeguards and cost justification.
